# As the World Struggles With the COVID-19 Pandemic, Another Emergency Threat Arrives on the Horizon, the Monkeypox: A Systematic Review

**DOI:** 10.7759/cureus.33596

**Published:** 2023-01-10

**Authors:** Satish Kumar, Kumar Rahul, Amit K Gupta, Harish Gupta, Satyendra K Sonkar, Virendra Atam, Shyam C Chaudhary

**Affiliations:** 1 Medicine, King George's Medical University, Lucknow, IND; 2 Cardiovascular and Thoracic Surgery, King George's Medical University, Lucknow, IND; 3 Medicine, King George’s Medical University, Lucknow, IND

**Keywords:** vesiculo-pustular rash, homosexuality, zoonotic, smallpox, monkeypox

## Abstract

The whole world got threatened by COVID-19, which made a significant loss in various sectors and pushed the world into a deep valley. Now a new threat, the emerging outbreak of monkeypox is rapidly spreading across the globe and is currently being observed in more than 110 countries with 79,473 confirmed cases and 50 deaths. Data were collected from PubMed, EMBASE, MEDLINE, Cochrane, Scopus database, African Journals OnLine, internet library sub-Saharan Africa, and Google Scholar. Most data were taken from the democratic Republic of Congo, the Central African Republic, Cameroon, the Republic of Congo, Liberia, Nigeria, the US, and the UK. Case reports, outbreak investigations, epidemiological studies, and surveillance studies were reviewed to find epidemiological details about the outbreak. A total of 50 peer-reviewed articles and 20 grey literature articles, including 9050 cases, were identified for data extraction. Our systematic review revealed that the group most affected is male (95.5%), with a median age of 33.8 years. A total of 55% of the transmission was sexually transmitted. The most commonly reported symptoms such as vesicular-pustular rashes (97.54%), fever (55.25%), inguinal lymphadenopathy (53.6%), exanthema (40.21%), fatigue, headache, asthenia (26.32%), myalgia (16.33%), vesicles and ulcers (30.61%) in the anogenital regions were some of the significant findings. The case fatality rate was observed to be up to 8.65%. The most affected country was the USA, which has the most fatalities in younger ages involved in homosexuality, suffering from HIV or sexually transmitted diseases (STDs).

## Introduction and background

**Figure 1:**
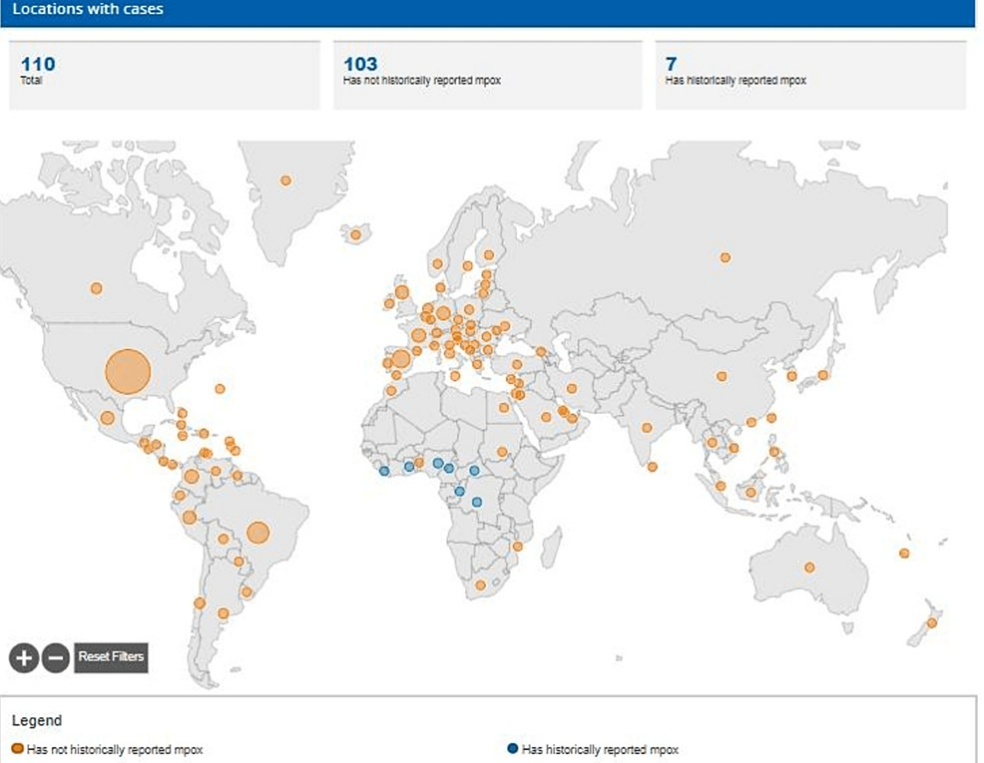
Map of countries affected by monkeypox.

COVID-19, or SARS-CoV-2, which allegedly spread from Wuhan city, China, in December 2019 (the first case on December 31, 2019, in Wuhan city), became a pandemic and stroke massive fatalities across the globe. After its first wave in 2019-2020, the second (March-June 2021), third and fourth waves were there, which kept people locked in their homes for several months, and even at present, it is running across the world. The whole world got threatened by COVID-19. It made a tremendous economic loss and pushed the world into a deep valley. A new health emergency threat, monkeypox, which was exclusively an African disease, is now spreading globally and involving several countries, pushing the world into an upcoming health crisis (Figure 1). The WHO declared monkeypox a public health emergency of international concern on July 23, 2022.

Monkeypox disease is a viral illness with maculopapular rash, similar to chickenpox. The virus was named monkeypox because of its very close similarity to other pox viruses. The monkeypox virus was first isolated in monkeys kept for research purposes in 1958 in Copenhagen, the capital of Denmark. Several outbreaks occurred in the United States and the Netherlands from 1960 to 1968 [1]. Monkeypox virus was first identified in humans in 1970 in a nine-month-old infant, with a pustular lesion similar to pustular eruptions of smallpox [1, 2]. The disease has three phases of progress: incubation, prodrome, and the eruptive stage.

Virology-monkeypox virus is a zoonotic orthopoxvirus that belongs to the family Poxviridae, subfamily Chordopoxvirinae, and the genus Orthopoxvirus [3]. The electron microscopic picture shows a rectangular or ovoid, brick-like shape. The virus is a large enveloped virus, with each virion encapsulating a core containing a linear double-stranded DNA genome of 200 kilobase pairs encoding about 200 proteins. The viral cycle exclusively takes place inside the infected cell. There are two lipoproteins envelopes: an internal envelope surrounding the capsid and an external envelope covered with viral surface proteins for adherence to the host cell. Smallpox virus variola is genetically closely related to monkeypox. Other viruses like cowpox, camelpox, and vaccinia belong to the same genus. There are four open reading frames in the inverted terminal-inverted repeats in the monkeypox genome, which are absent in vaccinia. Its codon usage indices indicate synonymous codon usage bias and selection pressure balanced by mutational pressure [4]. There are two genetic clades of monkeypox whose genome differs by less than 1%. The western African strain and the Congo basin strain are the two main genetic clades of the human monkeypox virus. Congo basin strain has higher morbidity, transmission, and mortality. The case fatality rate is approximately 1-11% in unvaccinated subjects. WHO renamed the groupings clade 1 and clade 2 (with subclade IIa and subclade IIb) so that geographic stigmatization can be averted. The current outbreak is considered due to the newer strain, clade 3, originating from the West African strain.

We conducted a systematic review by critically analyzing the published literature on monkeypox to review reported data, epidemiological variations, and its implementation for the control. The current study will benefit and provide valuable insight to healthcare providers and epidemiologists regarding the understanding of the monkeypox outbreak. As of now, a complete, consistent and long-term study is required to form and formulate guidelines to efficiently and effectively manage the disease.

## Review

Material and methods

Study Setting and Design

This systematic review was done per Preferred Reporting Items for Systematic Reviews and Meta-Analysis (PRISMA) guidelines.

Search Strategy and Study Selection

**Figure 2:**
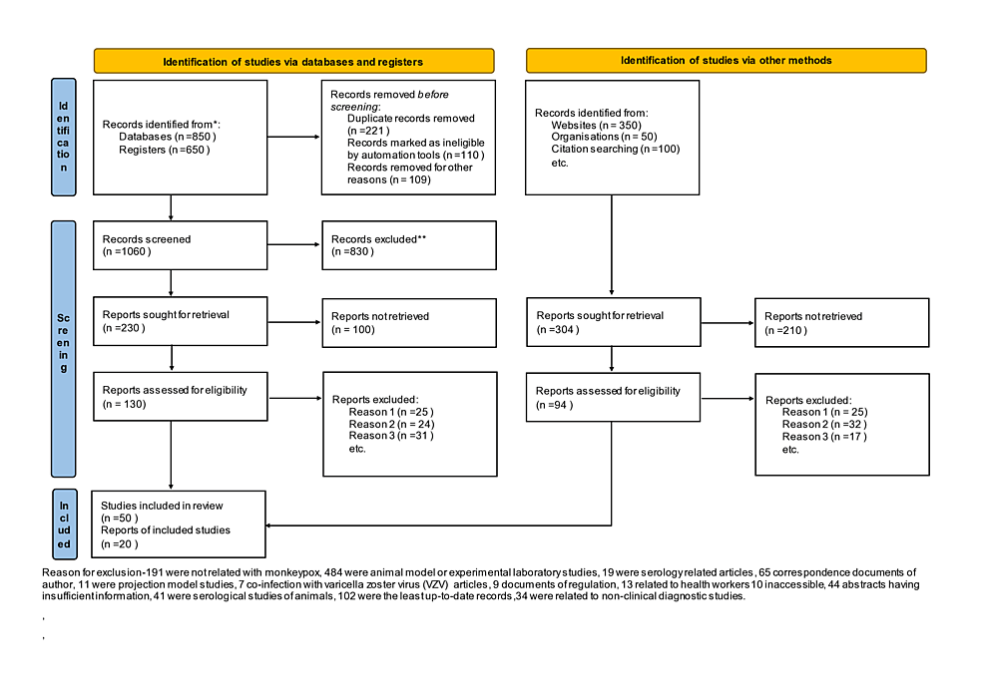
PRISMA flow chart for study selection. PRISMA: Preferred Reporting Items for Systematic Reviews and Meta-Analysis.

The present systematic review was conducted by using the keywords "monkeypox, monkeypox virus, monkeypox in USA, monkeypox in Africa, monkeypox outbreak, monkeypox and travel, monkeypox in Europe" in PubMed, EMBASE, MEDLINE, Cochrane, Scopus database, African journals online, internet library sub-Saharan Africa, Google Scholar, WHO Global Health Library (GHL), Virtual Health Library (VHL), Institute of Science Index (ISI), New York Academy of Medicine Grey Literature Report (NYAM), System for information on Grey Literature in Europe (SIGLE), and POPLINE and Cochrane Central Register of Controlled Trials (CENTRAL). In addition to these primary sources for literature, grey literature and Google sources like ProMED, epicenter, African Field Epidemiology Network (AFENET), websites of the WHO, US CDC, Africa CDC, and Nigeria CDC, a weekly bulletin on outbreaks, and other health emergencies were also used for data collection. Ministry of Health Africa and Google search sites were searched for information regarding outbreaks (Figure 2).

Inclusion and Exclusion Criteria

Studies related to epidemiological features of human monkeypox like case fatality ratio (CFR), transmission dynamics, case numbers, genetics of strains, studies concerned with surveillance data or case definitions, investigations for outbreak or situation reporting, risk factors for transmission studies, studies discussing the treatment of monkeypox were included in the current systematic review. While topics not focusing on monkeypox but on smallpox or other zoonoses, serological studies, non-clinical diagnostics studies, experimental studies (laboratory studies), and animal serological studies were excluded from the study.

Data Extraction and Analysis

Articles published in English from inception to October 2022 were considered for this systematic review. Recent information regarding multiple monkeypox outbreaks was selected and included in the study. After screening all selected articles, the review was done, and data of interest were fed into the data extraction sheet. Analysis and assessment were done for median age, CFR, geographical spread, and various newer signs and symptoms.

Results

The search included about 2000 publications related to monkeypox, of which 100 were fully screened, and 70 of them were used for data extraction.

Monkeypox outbreaks

Outbreaks in African Nations

Its outbreaks were seen in 1980 in the Democratic Republic of the Congo (DRC) and from 2017 to 2018 in the DRC, Central African Republic (CAR), Cameroon, Republic of Congo, Liberia, and Nigeria. WHO announced that "endemic monkeypox has been reported recently from more countries in the past decade than during the previous 40 years". The mean incubation period is about 13 days (range 3-34 days) [5,6]. One to four days of the prodromal phase is characterized by high temperature, headache, fatigue, and lymphadenopathy, especially in cervical and maxillary regions. Lymphadenopathy is a distinguishing point from chickenpox. The eruptive phase lasts for 14-28 days and is characterized by centrifugal development of deep, well-circumscribed, macular, papular, vesicular, pustular, and lastly, crusted scab lesions [3-6]. Frequent development of lesions on the palm and soles differentiates it from chickenpox. In rare cases, genitals, oral ulcers, and conjunctiva damage (corneal ulcerations and vision loss) may occur. The severity of monkeypox was seen in pregnant women and children. Immunocompromised individuals have higher chances of getting infections and mortality following such infections [6-11]. Monkeypox has a self-limiting course, but post-viral sequelae may include pitted facial scars. In Nigeria, the CFR was 6%, and in DRC, it was 10-15%. Chickenpox is the main differential, and it may have a co-infection with monkeypox, as found between 2009 and 2014 in DRC, which was the most affected African country. Sexual transmission was observed rarely.

Current Outbreak

In the current multi-country outbreak, the first case/index case was reported on May 6, 2022, in the United Kingdom by a traveler from Nigeria. The WHO and other public health institutions raised the alert on May 16, 2022, and declared a global health emergency on July 23, 2022. As per WHO, by November 2022, global monkeypox outbreak cases are approximately 79,473 in more than 110 countries. The current number of cases in the US is 52,272; the European region 25,375; the African region 958; the Western pacific region 216; the Eastern Mediterranean region 72; and the South-East Asia region 31. Among all cases, 78,500 were from locations that have not historically reported monkeypox. In the current outbreak, the vast number of monkeypox cases was among homosexuals/men sex men (MSM) (90.9%), bisexuals (6%), or have direct contact with virus-carrying individuals. Transmission by blood transfusion has not been reported, while semen carrying viral DNA was reported in one case report. Sexually transmitted infections (STIs) and HIV infections (52.6% of all HIV) are highly prevalent in monkeypox patients during the current global outbreak. A total of 30.7% of patients in the UK had more than ten sexual partners in the last three months. Monkeypox DNA has been detected in the seminal fluid of diseased males, but it cannot be considered evidence of infectivity. Male predominance was there (95.5%), with a median age of 33.8 years (interquartile range [IQR]: 29.55). Isidro J et al., in the current epidemic, performed phylogenomic characterization of the first monkeypox viral genome sequence. The mean incubation period was nine days. In addition to features in outbreaks of African countries, in the current outbreak, skin lesions were unusually found in the genitals and anal region without centrifugal occurrence. Rectal pain, penile edema, cellulitis, severe angina, epiglottitis, and ocular edema were the main causes of admission. The CFR was observed at less than 1%.

Changing/Shifting epidemiology of monkeypox

Monkeypox has always been a global and grossly neglected public health problem in different regions of Africa for decades, and now its spread has led to its reporting worldwide since May 2022. Most of the available data are from DRC and are limited to lower age groups. More than 80% of patients were observed in 1970-1979, 1981-1986, and 1996-1997 pediatric population [3,12]. In recent years, it has spread beyond the regional boundaries of Africa to many other countries. Homosexuality is the leading transmission mode as of now. During the past six months, monkeypox has spread to around 110 countries, leading the WHO to mark this outbreak as the highest level of alert. Increased surveillance and detection of infected patients are essential tools for finding the continuously varying epidemiology of monkeypox.

Transmission/Mode of spread of the monkeypox virus

**Figure 3:**
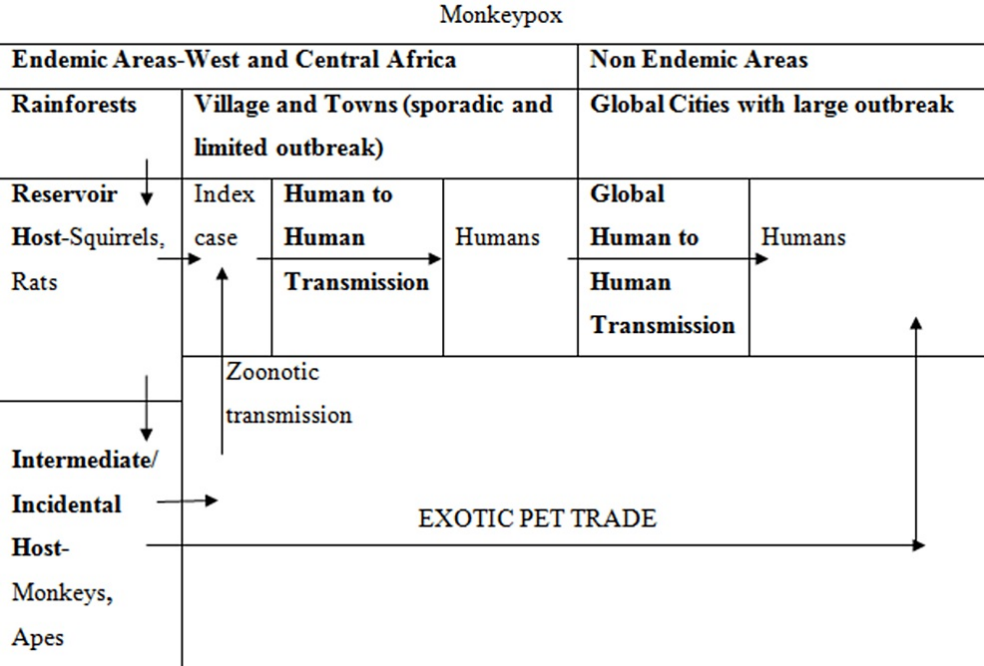
Schematic representation of the natural history of monkeypox.

Although it is a zoonotic disease, its animal reservoir remains unknown. Tree squirrels and Gambian pouched rats are strongly suspected candidates. African apes and monkeys are considered intermediate hosts (Figure 3).

Animal-to-Human Spread 

It may be by body fluids, direct blood contacts, and cutaneous or mucosal lesions of diseased animals. This systematic review noted a total of 10 reports where an animal source was observed. Four case reports could not report any animal source of infection, while 10 others did not observe any animal sources. Four reports stated that hunting, eating, and handling works are implicated in the primary infection of the disease. Cane rats, squirrels, and bemba were implicated in the CAR [13-15]. Sleeping on the floor, preparing and cooking wild animal meat, and eating duiker meat were reported in primary cases in DRC [3,16]. Animal rearing, contact with animal excreta, invasive contact with animals, and hunting or cooking wild animals was not significantly found to be associated (P=0.683). Some authors also reported that sharing pots by young males seems implicated in inter-human transmission, but there is a lack of evidence to support this hypothesis.

Human-to-Human Spread

Skin lesions, respiratory secretions of an infected human, or recently contaminated material may be a source for the spread. Transmission by respiratory droplets needs enough time for face-to-face contact, as in the case of healthcare workers and household members [3]. The secondary attack rate (SAR) range suggests that 0-11% of unvaccinated contacts of primary cases can be converted into clinical cases in an outbreak. One of the studies reported that the reproduction number (R0) or the secondary cases might arise due to a single primary case. It was found that the basic reproduction number was 0.8 during the outbreak of DRC, while the net reproduction number was 0.3 cases [17]. McMullen CL et al. observed a net reproduction number of 0.6 in DRC in the years 2005-2007 [18]. In DRC, 2001-2013, the CFR was found to be less than 5%, while in the Nigeria outbreak, it was 2.8%. CFR was found to be 0% in the outbreak in the USA, where 47 cases were reported. The leading aetiologies behind case fatality were mainly high-risk pregnancy, pediatric age group, and immunocompromised patients [19-21].

Clinical features of monkeypox

McCollum AM and Damon IK reported the case definition of DRC ministry in endemic areas as "a case with 'febrile prodrome with any of the (a) pustular rash or scars or vesicular on the face, palms, and soles, or (b) the presence of five or more variola-like scar" [19]. A total of 99% of sensitivity and 78% specificity was found when applied to clinical symptoms for four suspected cases, whose laboratory values were significant. A study done by Osadebe L et al. defined the case definition as "Individuals who have a vesicular or pustular eruption with deep-seated, firm pustules along with either of one a) febrile prodrome, b) lymphadenopathy (cervical, axillary or inguinal), and/or c) crusted pustules on the feet soles or hands palm" [22].

More than 78% sensitivity was found with clinical symptoms like fatigue, lymphadenopathy, and rash characterized by deep-seated, same-size lesions present on the legs, arms, soles, and palms. Conjunctivitis, nausea, and lesions on the genitals were characteristic features with 76% specificity and were found to occur in only 28% of cases. Various symptoms in decreasing orders are the development of rash, fever, malaise, chills, headache, myalgia, lymphadenopathy, pruritis, rectal pain, rectal bleeding, nausea, vomiting, abdominal pain, tenesmus, pus, proctitis, and conjunctivitis (Table 1) [23-24].

**Table 1:** Table showing original and newly proposed taxonomies and epidemiological features of three clades of the monkeypox virus. DRC: Democratic Republic of the Congo; CAR: Central African Republic; USA: United States of America.

Name of the Original Clades	Revised Nomenclature of Clades	Geographic Distribution of Clades	Epidemiological Characteristics of Clades
Central Africa Clade	1	DRC, Gabon, Cameron, CAR, South Sudan, Congo	Endemic disease, More sporadic cases, Outbreaks happen
West Africa Clade	2 (IIa and IIb)	Nigeria, Ivory coast, Cameron, Leone, Liberia, Sierra	Endemic disease, More sporadic cases, Outbreaks happen
New Form West Africa Clade	3	Europe, USA, Pacific regions, Middle east	Current pandemic with global occurrence

Novel presentations

Rectal Pain and Penile Swelling

It is observed that symptoms of monkeypox are now significantly different in comparison to earlier/previous outbreak symptoms in Africa. Newer symptoms include rectal pain and penile swelling [25]. This finding was based on a retrospective study of 197 patients positive for monkeypox in London, UK. A total of 196 out of 197 had a history of a gay relationship, bisexual or other men who had sex with men. 

Neurological Damage and Psychiatric Problems

A recent publication in the journal of e-clinical medicine suggests that two to three percent of monkeypox patients may develop serious neurological issues like headaches, seizures, and encephalitis. Anxiety, depression, and confusion were also observed (Table 2) [24].

**Table 2:** Characteristics of old and newer forms of the monkeypox virus. USA: United States of America; MSM: Men sex men; CFR: Case fatality ratio.

Defining variables	Old form, 1970 to the present	Newer form, 2022
Country	Endemic countries like Central and West Africa	Non-endemic regions like Europe, the USA, Middle east Australia
Age group	Pediatric group and young adults	MSM with young age (31-40 years)
Epidemiological features	Cases are sporadic and epidemics	Current outbreak (pandemic underway since May 2022)
Mode of transmission	Infected animal reservoir contact, then human-to-human transmission	Only human-to-human transmission
Dissemination	Interfamilial in most cases	In sexual contact, unprotected sex with multiple male partners
Phases of presentation	Incubation phase, prodromal phase, eruption phase	Incubation phase, prodromal phase (may be absent), eruption phase with unusual distribution of lesions especially in genitals
Symptoms of monkeypox	Centrifugal distribution of lesions on face and extremities along with or without cervical or axillary lymphadenopathy	Novel presentations like penile edema and rash, vesicular and ulcerative lesions, perianal rash, proctitis/rectal pain, pharyngitis, tender inguinal lymphadenopathy, neurologic and psychiatric problems
Clades of viruses	Clade 1 (Central African Clade) and Clade 2 (West African Clade)	Clade 3 (West African Variant)
CFR	1-15 %	0.025 (Less than 1%)

Etiology of monkeypox infection

The regions prevalent with previous outbreaks have animals like monkeys, rodents, squirrels, bunnies, gazelles, and porcupines. People involved with butchering animals, catching, and eating flesh in forests were prone to the disease because of higher chances of contamination. A trial for monkeypox infection in DRC in 1997 was done on various wild creatures like Gambian rodent (*Cricetomysemini*), homegrown pig (*Susscrofa*), Kuhl's tree squirrel (*Funisciuruscongicus*), sun squirrel (*Heliosciurusrufobrachium*), elephant wench (*Petrodromustetradactylus*), and Thomas' tree/rope squirrel (*Funisciurusanerythrus*) where it was found that they have antibodies against monkeypox, means are normal repositories. This replaced the unmistakable quality of creature-to-human transmission [26,27].

Diagnostic tests

Clinical evaluation rests on differentiating features common with other diseases like measles, syphilis, chickenpox, scabies, drug allergies, and bacterial skin infections. Lymphadenopathy in monkeypox differentiates it from smallpox and chickenpox. Using the FDA 510(k)-cleared non-variola orthopoxvirus (NVO) real-time polymerase chain reaction (PCR) test and laboratory-developed real-time PCR tests, suspected cases can be diagnosed. PCR assays (NVO and other orthopoxvirus laboratory-developed tests [LDT]) are the main tools for diagnosing the disease. Molecular tests like real-time PCR are very specific as well as sensitive. According to the CDC, re-extraction and retesting of the specimen should be done in case of an absence of epidemiological criteria and a high Ct value (>34). A biopsy is also an option [28].

Sampling

Wear personal protective equipment (PPE) kit before taking the sample, two swabs from each lesion and from different body parts, and put into two different virus transport media (VTM). The sample must be a dry, swabbed, or lesion crust, and put in a VTM vial. Specimens are to be sent first to the laboratory response network (LRN) or an authorized commercial laboratory.

Shipment of the Sample

Within an hour of collection, the sample should be stored and refrigerated (2-8°C) or frozen (-20°C or lower). Shipment of samples should be done on dry ice, if possible. If any sample is received outside acceptable temperature ranges, it should be rejected. An electronic Global File Accessioning Template (GFAT) form can be used to ensure that each sample is labeled with a unique identifier GFAT.

Management of monkeypox

The fatality is about 1-10% in African cases of monkeypox, and in other cases, it is generally a self-restricted infection. In the new flare-up of monkeypox infection in the USA, fatality is low. The patient complains of fatigue/lassitude in the febrile phase, and bed rest is indicated. In critical cases, hospital admission is crucial. Airborne and contact safety measures should be taken care of to avoid infection to medical care and other close contacts. According to recent CDC guidelines, monkeypox separation should be done until the last outside layer is shed.

Prevention 

Education and awareness for self-protection: Education and awareness among the general population about preventive measures and risk factors are of utmost importance. Various studies are in the run to have the feasibility and appropriateness of prevention and control of monkeypox with vaccination. One should avoid close contact and maintain at least six feet distance [24,26].

Safer sex strategies: The WHO Director-General said that from May 2022 till now, among all reported cases, the maximum is having acts of MSM, gay, and bisexuality. If the partner has any monkeypox symptoms on the body, including the mouth, genitals (penis, testicles, vulva, or vagina), or anus (butthole), then avoid sex of any kind (oral, anal, and vaginal) and kissing or touching partners. Towels, fetish gear, sex toys, and toothbrushes should not be shared. Reducing the number of sexual partners and avoiding spaces like sex clubs, back rooms, saunas, or private and public sex parties are recommended [28].

Preventing spread to others: As per the WHO, monkeypox infects anyone in close contact with a patient or their contaminated clothing or bed sheets, direct contact, skin-to-skin contact, possibly even face-to-face contact, making exposure to droplets. That is why isolation is an issue [28]. The patient should not share things that he has touched and should stay away from pets, livestock, and other animals. Patients should also not share bathrooms, towels, washcloths, toothbrushes, or drink from the same glass. The patient should avoid public transit, avoid ride-share services, should wear a mask, and infected places should be disinfected.

Intriguing creatures: Monkeypox patients should avoid contact with animals, including domestic animals, pets, and wildlife [28].

Restrictions on animal trade: Some countries regularly import non-human primates and rodents; it should be restricted because these animals are reservoirs of monkeypox viruses. Along with this restriction, infected animals should be immediately isolated and quarantined with others [28].

Vaccination: Vaccine for the prevention of smallpox and monkeypox - the JYNNEOS vaccine has been approved. During the current outbreak of monkeypox in the USA, JYNNEOS is the primary vaccine being used. The ACAM2000 vaccine is the next alternative to JYNNEOS, which is approved for protection against smallpox and monkeypox. Now many viral infections can be prevented by vaccines, and immune imprinting is widely studied for them. Immune imprinting is a phenomenon when primary exposure is confined to the activated B-cell memory to one strain, limiting from producing memory B-cells and neutralizing antibodies for other novel variants.

Treatment

Currently, there are no specific treatments for monkeypox disease. As monkeypox and smallpox viruses are genetically identical, antiviral drugs and vaccines developed for smallpox can also be used to prevent and treat monkeypox virus infections. Vaccinia-invulnerable globulin (VIG) is unsuitable for prophylaxis and treatment [29]. ST-246 is a concentrate prepared from the monkeypox infection contamination of the creature model, which was found to have adequacy for prophylaxis, post-exposure, and restorative treatment of monkeypox. Antibody for smallpox and monkeypox (JYNNEOS), which is a weak, nonreplicating, and live vaccine, was supported by the US FDA in September 2019 for post-exposure prophylaxis and is also used in the current outbreak in the US [27,29]. Clinical care should be fully optimized so symptoms, complications, and long-term sequelae can be alleviated. Food and fluids to maintain adequate nutritional status should be administered. Antibiotics can be used to treat secondary bacterial infections [29].

Antiviral Agents

Ticovirimat: Tecovirimat (TPOXX), an antiviral agent used for smallpox, is now licensed for treating monkeypox by European Medicines Agency (EMA) in 2022 after taking the informed consent form, patient intake form, FDA form 1572, and serious adverse event reporting. This agent is not yet widely available [30]. This is only for those with severe diseases like lesions that have merged into larger lesions, infected or bleeding lesions, or high-risk patients, patients with weakened immune systems or skin conditions like HIV, eczema, lymphoma, leukemia, on chemotherapeutic agents, organ transplantations, children with autoimmune diseases, and pregnant, breastfeeding mothers. TPOXX prevents or minimizes severe monkeypox disease that involves the mouth, genitals, eyes, anus (butthole), and throat. TPOXX relieves short-term symptoms like pain, abscesses, and swelling and long-term effects such as scarring. In case of milder monkeypox symptoms, TPOXX should be avoided.

Cidofovir: The drug of choice used till now is Cidofovir for critically ill cases. This is a nucleotide analog molecule that was found to be selectively inhibiting the replication of herpes and CMV viruses [26]. Active immunity by generating antibodies against the smallpox virus is created by the vaccinia vaccine. Smallpox prevention and treatment are indicated just after the infection or any sign of infection by primary immunization. US military personnel, health care professionals, and US Department of Defence civilian employees have indicated personnel to get this vaccine as they may be a case for bioterrorism.

Smallpox vaccine: As per the CDC, a smallpox immunization for laborers uncovered medical services and family contacts of confirmed cases should be done within 14 days of openness, preferably within four days. Smallpox, vaccinia, molluscum, and monkeypox belong to a similar family. Smallpox was eradicated from the world in 1979-1980. The smallpox vaccine was prepared from the live vaccinia virus, not killed or weakened. The smallpox vaccine is effective at up to 85% against monkeypox disease. Those individuals who have prior smallpox vaccination may have a mild illness.

Now there is a newer vaccine, the modified attenuated vaccinia virus (Ankara strain), which was approved as preventive therapy for monkeypox in 2019 [26]. Vaccinia immunoglobulin, purified plasma gamma globulins, NIOCH-14, ACAM 2000, MVA-BN, LC16m8 vaccines are under trial.

Discussion

Monkeypox in humans has been reported in at least 11 countries of Africa after 1970, including Cameroon, the DRC, Benin, Gabon, Liberia, Cote d'Ivoire, Nigeria, Sierra Leone, South Sudan, CAR, and the Republic of the Congo. A large outbreak occurred in 1970 in Nigeria, with 200 confirmed and more than 500 suspected cases with a CFR of 3%. Monkeypox has now become a disease of global public health importance. After 2003, when the first case was found outside Africa, many more cases beyond African countries were found. It was in the US in infected pet prairie dogs from Gambian pouched rats and dormice imported from Ghana. After that, more than 70 cases were reported, and an outbreak was declared. Travelers from Nigeria to different countries were also reported to be monkeypox positive, including the UK in September 2018, Singapore in May 2019, Israel in September 2018, December 2019, May 2021, and May 2022, and the USA in July 2021 and November 2021. Many other non-endemic countries also reported cases of monkeypox in May 2022 [31,32]. Further studies are required to understand the pattern of transmission, infection sources, and epidemiology. 

This systematic review enables us to understand epidemiology and pathophysiology since it was initially detected in humans in 1970. In the initial year, 2000, suspected cases increased in DRC, reaching>10,000 cases in the years 2000-2009 to>18,000 in the years 2010-2019 [33-35]. DRC alone reported at least 4,594 suspected cases in 2020, and it reached 6,257 up to the end of the year [33]. In Nigeria, confirmed cases increased from 3 in 1970 to 181 in 2017-19 [34]. In DRC, it ranged from 511 confirmed cases in 1990 to more than 28,000 in the years 2000-2019. Hoff NA et al. observed that the increase in cases in the DRC from 2001 to 2013 was the actual rise and not because of improved surveillance [35]. During the previous five decades, its outbreak was observed in more than 10 nations in Africa and at least four countries outside Africa. After the re-emergence of the disease in Nigeria in the years 2009 and 2010, monkeypox patients were also found to emerge in Sierra Leone and Liberia after four decades. The Republic of the Congo reported its initial outbreak in the years 2000-2009 and in South Sudan in 2005. Outside Africa, monkeypox patients have been found since 2003. The USA got the infected rodents from Ghana, but Ghana itself has not reported any human cases. The animal-to-animal transmission then led to animal-to-human transmission, which resulted in the outbreak of 47 cases. Starting from the year 2018 up to 2022, people traveling from Nigeria, the USA, Singapore, the UK, and Israel tested monkeypox positive. They were suspected to be due to animal-to-human transmission [36]. Index cases may be those who are infected with the virus and are responsible for newer transmission. This was seen in the UK and the US in 2021 [37]. Human-to-human transmission of the disease was significantly observed in the Republic of the Congo, Nigeria, South Sudan, DRC, and CAR [38-41]. These transmissions indicate that both clades are susceptible to human-to-human transmission. The R0 value indicates the epidemic potential, which is more than 1 in monkeypox [42]. The waning of immunity is one of the essential resurgence factors. Previously, when smallpox was dominant, monkeypox cases were nil. This may be because of people's focus on smallpox and not on monkeypox, as both share common presentations, and 85% of cases were treated with a similar vaccine [43]. Routine smallpox vaccination was ceased as it was declared eradicated in 1980 by the World Health Assembly [44]. According to the observations of Grant R et al., in 1970, about 65.6% of people were vaccinated for smallpox, while in 2016, this vaccination decreased to 10.1%, which shows a waning of immunity. Up to 2018, the strength of the vaccinated population decreased to 9.3%, and an overall estimated immunity reduced to 2.2%. In this review, we observed that approximately 97% monkeypox positive patients were unvaccinated for smallpox. One reason for the resurgence of the disease may be a genetic modification of the monkeypox virus. We analyzed those young children in the age group of up to five years were more affected in 1970-1989, and in 2000-2009 this age group increased to 10 years, and it was 21 years in 2010-2019 to 33.8 years in 2022. The CFR was 100% in the initial years in the pediatric age group, while it reduced to 37% in the years from 2000 to 2022.

## Conclusions

The geographical spread of monkeypox disease is now a global issue. The present systematic study has reviewed old and current outbreak studies. Monkeypox was an endemic disease in Africa with a similar presentation and severity to smallpox, as both viruses belong to the same family. Gradual decline in immunity to smallpox may be an explanation for frequent outbreaks. Now the disease is affecting primarily men of the younger age group involved in homosexuality. The clinical scenario of monkeypox is changing. There is insufficient knowledge about monkeypox, and as of now, the general population is less aware of it. Healthcare providers should take responsibility for its awareness, education for high-risk groups, development of rapid, sensitive tests, and to evaluate the effectiveness of available treatments and vaccines.
